# Effective Technique and Mechanism for Simultaneous Adsorption of As(III/V) from Wastewater by Fe-ZIF-8@MXene

**DOI:** 10.3390/toxics12060419

**Published:** 2024-06-07

**Authors:** Shuyan Zang, Qing Zhang, Baoli Hu, Yaqian Zhang, Jaan H. Pu, Meiheng Lv

**Affiliations:** 1 College of Science, Shenyang University of Chemical Technology, Shenyang 110142, China; zangshuyan@126.com (S.Z.);; 2 Faculty of Engineering and Digital Technologies, University of Bradford, Bradford BD7 1DP, UK

**Keywords:** kinetics, thermodynamic, adsorption, arsenic, Fe-ZIF-8@MXene, actual wastewater

## Abstract

Arsenic (As) contamination of surface water has become a global concern, especially for the third world countries, and it is imperative to develop advanced materials and an effective treatment method to address the issue. In this paper, iron doped ZIF-8@MXene (Fe-ZIF-8@MXene) was prepared as a potential adsorbent to effectively and simultaneously remove As(III/V) from wastewater. To investigate this, Fe-ZIF-8@MXene was characterized before and after the removal of mixed As(III/V). The results of Fourier transform infrared (FTIR), X-ray diffraction (XRD), scanning electron microscopy (SEM), transmission electron microscopy (TEM), X-ray photoelectron spectroscopy (XPS), specific surface area (BET) and point of zero charge (pHpzc) showed that Fe-ZIF-8@MXene was prepared successfully and kept a stable structure after As(III) and As(V) adsorption. The particle size of Fe-ZIF-8@MXene was in the range of 0.5 μm to 2.5 μm, where its BET was 531.7 m^2^/g. For both contaminants, adsorption was found to follow pseudo-second-order kinetics and was best-fitted by the Langmuir adsorption model with correlation coefficients (R^2^) of 0.998 and 0.997, for As(III) and As(V), respectively. The adsorbent was then applied to remove As from two actual water samples, giving maximum removal rates of 91.07% and 98.96% for As(III) and As(V), respectively. Finally, removal mechanisms for As(III/V) by Fe-ZIF-8@MXene were also explored. During the adsorption, multiple complexes were formed under the effect of its abundant surface functional groups involving multiple mechanisms, which included Van der Waals force, surface adsorption, chemical complexation and electrostatic interactions. In conclusion, this study demonstrated that Fe-ZIF-8@MXene was an advanced and reusable material for simultaneous removal of As(III/V) in wastewater.

## 1. Introduction

Arsenic (As) has become a worldwide concern because it is a carcinogenic trace metal [1,2]. Long-term exposure to As-containing substances is strongly linked to many types of cancer, such as skin, bladder, lung and respiratory tract cancers, and extensive liver damage [3,4]. Many developing countries and poor areas have been overexposed to As, with high As concentrations in their water supplies due to low management ability and public awareness, and inadequate water treatment [5,6,7]. It was reported that groundwater pollution seriously threatens the health of nearly 100 million of the population in India [8]. Underground thermal activity in some countries has resulted in a high concentration of As in geothermal water, i.e., in Japan, ranging from 1.8 to 6.4 μg/mL, whereas the level of As in New Zealand could reach up to 8.5 μg/mL [9,10]. The World Health Organization (WHO) and the United States Environmental Protection Agency (EPA) have imposed a maximum concentration of less than 10 μg/L in drinking water [5]. Therefore, in order to meet water standards in all walks of life, an effective As removal technology and mechanism is in demand.

To adequately remove all kinds of the As from water sources, lots of effective methods and materials have been studied, including adsorption [11], chemical co-precipitation [12], ion exchange [13] and biological treatments [14]. Among these methods, adsorption is considered as one of the most promising techniques, with a high performance-to-price ratio [8,15]. However, the adsorbents used in the past only possessed limited characteristics, typically one or two. It is particularly important to simultaneously possess multiple advantageous adsorption characteristics, especially those with specialized properties for adsorbing arsenic. A good adsorbent should have low solubility, not produce secondary pollution materials and provide a large specific-surfacearea and porous structure [16,17,18,19]. Metal–organic frameworks (MOFs) have attracted considerable interesting because of their wide applications in wastewater treatment, based on their large BET, high porosity, adjustable pore size and strong thermal stability [20,21]. Recently, several kinds of MOFs, like the University of Oslo’s Materials Institute Lavoisier and ZIFs (zeolitic imidazolate frameworks), especially ZIF-8, have been suggested to the public based on their importance for removing various contaminants, including As, from wastewater [22,23]. However, the solid–liquid separation problem of MOFs after adsorption has limited their use in real-world applications.

To solve this problem, MOF-based composites with Fe have been proven to be a potent method [24,25]. Due to the high affinity between As and iron, the adsorbent containing iron species would enhance the adsorption rate of As(III) and As(V), and further transform it into stable compounds, which is more beneficial to practical applications [26]. The mobility and fate of As(III) and As(V) are highly dependent on the content of iron (hydr) oxides from adsorbent, which are one of the most important sinks for As by the formation of insoluble FeAsO_4_ [27]. Environment-friendly two-dimensional MXene (Ti_3_C_2_T_x_) material has a large number of active sites and rich functional groups, which can effectively adsorb heavy metal ions in water [28,29,30]. Compared with other MOFs, ZIF-8@MXene exhibits higher thermal and chemical stabilities [31]. The heterostructures integration of ZIF-8@MXene could be prepared by the chemical complexation and physical deposition of metal (oxide) into pores. Zou et al. [32] suggested Fe_3_O_4_@ZIF-8 gives significant improvement to As adsorption by qualitative and quantitative analysis. To date, although many MOF-based composites have been reported for adsorption of As(III) and As(V) [33], there is still a lack of studies on a single material which simultaneous combines several special metals and functional groups together to give high efficiency removal of As(III) and As(V) without secondary pollution.

The main objectives of this study were (1) to prepared an adsorbent simultaneously possessing low solubility, with no secondary pollution, large specific surface area and porous structure; (2) to investigate the adsorption kinetic and isotherm behavior of Fe-ZIF-8@MXene on As(III) and As(V); and (3) to tentatively explore the main interaction mechanism between As species and adsorbent.

## 2. Materials and Methods

### 2.1. Materials Preparation

In this study, all utilized chemical reagents were of analytical grade and were used without further purification. Primary concentrations of As(V) and As(III) solutions, each of them with 1000 mg/L, were purchased from Sigma-Aldrich (Germany). The fresh As(III) and As(V) working solutions were prepared by diluting the stock arsenate solutions (stored in the dark at 4 °C) with deionized (DI) water. The concentration of the adsorbate is based on the weight of As. All the glassware was soaked in 10% HNO_3_ for 24 h and cleaned 2–4 times with DI water before being used.

### 2.2. Synthesis of Materials

#### 2.2.1. ZIF-8 Synthesis

Firstly, 3.81 g 2-methylimidazole(C_4_H_6_N_2_) and 1.47 g zinc nitrate [Zn(NO_3_)_2_] were dissolved in 50.0 mL methanol, respectively. After 15 min of ultrasound, those two bottles of solution were poured into the same conical bottle for mixing. Ultrasound was then performed again for 10 min. The mixed solution was placed into the preheated thermostatic water bath with magnetic stirring, at 30 °C and 150 r/min, for 5 h. Then, the suspended products were collected by centrifugation with high speed and carefully washed 2–3 times with methanol. The fresh products were dried in an oven at 700 ± 5 °C for 5 h and then kept in a desiccator for the following experiments.

#### 2.2.2. Preparation of Fe-ZIF-8@MXene

Three substances, ZIF-8 0.50 g, MXene (Ti_3_C_2_T_x_) 0.10 g and Fe (NO_3_)_3_ 0.50 g, were put into 50.0 mL methanol to dissolve, then underwent 15 min of ultrasound at room temperature (the bottle was sealed with plastic wrap to prevent the evaporation of methanol during ultrasound). After that, the above mixed solution was placed into the preheated thermostatic water bath with magnetic stirring, at 25 °C and 150 r/min, for 45 min. Next, the water bath temperature was raised to 90 °C, and the methanol was gradually dried. The obtained solids were placed into the oven at 60 °C for drying, where the iron modified ZIF-8@MXene (Fe-ZIF-8@MXene) samples were obtained.

### 2.3. Real Water Samples

Two kinds of real water samples were used in this study. One was the river water (RW) collected from the Hunhe in Shenyang city, Liaoning Province, China. The other was the treated wastewater collected from the secondary settling tank in Shenyang Xihe municipal Wastewater Treatment Plant (WTP) Water samples were stored at 4 °C before performing 54 experiments.

### 2.4. Batch Adsorption Experiments

To investigate the adsorption ability of Fe-ZIF-8@MXene for mixed As(III) and As(V) pollutants, batch experiments were carried out. The coexisting systems of As(III) and As(V) (1–10 μg/mL) were designed to investigate the competitive adsorption between As(III) and As(V) at 15–40 °C with sequential dosed adsorbent. Lastly, to explore the effect of pH on the adsorption efficiency of As(III) and As(V) the solution’s pH was adjusted at intervals to desired pH ranges (from 4.0 to 9.0) with 0.1 mol/L NaOH or HCl.

### 2.5. Adsorption Kinetics of As(III) and As(V) by Fe-ZIF-8@MXene

The equilibrium time of As(III/V) adsorption was measured by the following experiments. An amount of 0.1 g adsorbent was placed into a conical flask with 100 mL different initial concentrations C_0_ (1.0, 2, 3, 5 and 10 μg/mL) of As(III) and As(V), and then the conical flask was stirred on a shaker (150 rpm), and the solution pH was 5.8. The sampling time range was 5 min–48 h, respectively. To determine the residue concentrations of As(III) and As(V), 1 mL sample solution was taken at different interval times, then filtered with 0.22 μm filter. The computed method of removal or adsorption rate (R, %) and the removal (or adsorption) capacity (*q_e_*, mg/g) of As(III) and As(V) were according to the following Equations (1) and (2):

(1) $$R=\frac{\left(C_{0}-C_{e}\right)}{C_{0}}\times 100\%$$

(2) $$q_{e}=\frac{V(C_{0}-C_{e})}{m}$$

where *C*_0_ and *C_e_* are the initial and equilibrium concentrations of As(III) and As(V) (μg/mL), *V* is the solution volume (L) and *m* is the mass of Fe-ZIF-8@MXene (g).

### 2.6. Adsorption Isotherms

To explore the maximum adsorption capacity of Fe-ZIF-8@MXene, the adsorption isotherms for the adsorbent were conducted. Initial concentrations of As(III) and As(V) were 1.0, 2, 3, 5 and 10 μg/mL, respectively, with utilization of 0.1 g/L Fe-ZIF-8@MXene shook at 150 rpm for 4 h, where the pH was maintained at 5.8. The Langmuir and Freundlich isotherms were obtained from the test results under the optimal condition.

### 2.7. Analytical and Characteristic Methods

As(V) was first converted into As(III) by reduction reaction (with double reducing agent: 10 g CH_4_N_2_S and 10 g ascorbic acid in 200 mL H_2_O), and then qualitative and quantitative analysis was performed by atomic fluorescence spectrophotometer (AFS-2202E, Beijing, China). The concentration of As(V) was calculated by subtraction, as proposed by Zang et al. [34]. The morphology of Fe-ZIF-8@MXene was characterized and analyzed by X-ray diffraction (XRD, RINT2000, Japan), Fourier transform infrared (FTIR, NICOLET6700, Thermo Fisher Scientific, USA) and scanning electron microscopy (SEM, S-3400N, Hitachi, Japan). For the Brunauer–Emmett–Teller (BET) specific surface area, transmission electron microscopy (TEM) was performed on a JEM-200CX electron microscope (JEOL, Japan) and a Tecnai 12 electron microscope (Philips, The Netherlands). X-ray photoelectron spectroscopy (XPS) spectrum was recorded on PHI 5000 VersaProbe (Ulvac, Japan), pore size distribution and pore volume of the adsorbent were performed by a N_2_ adsorption–desorption isotherm analyzer (ASAP 2010M, Micrometritics, USA). The zeta potential-pH of the Fe-ZIF-8@MXene was quantified based on the method described by Wang et al. [35].

## 3. Results

### 3.1. Characterization and Evaluation of the Adsorbent

**Figure 1:**
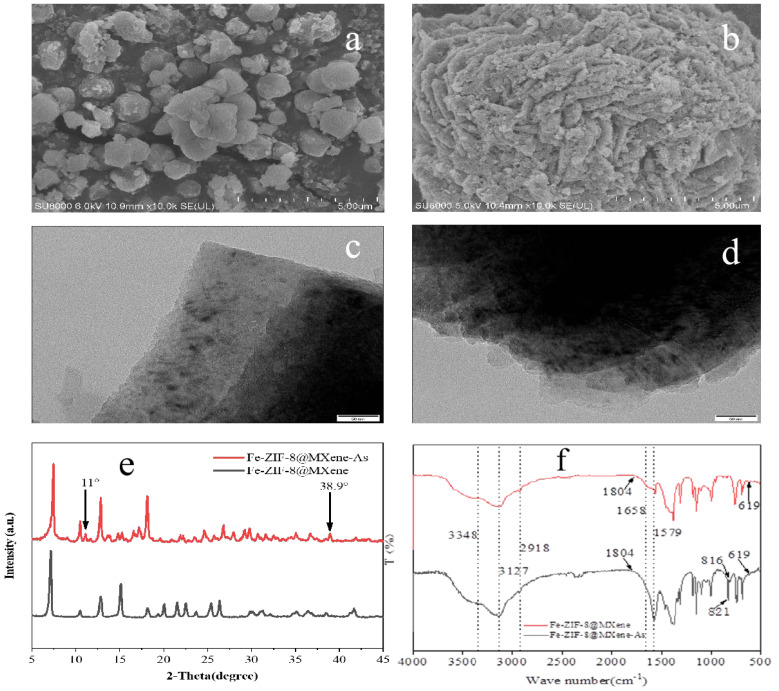
Spectrum characterization of Fe-ZIF-8@MXene. (**a**) and (**b**) are SEM images before and after adsorption, respectively, (**c**,**d**) are TEM images before and after adsorption, (**e**) is XRD image, and (**f**) is FTIR image.

The morphology of Fe-ZIF-8@MXene before and after the treatment of As(III) and As(V) in wastewater was characterized by SEM and TEM. As seen in Figure 1a,b, before exposure to the contaminant mixture, Fe-ZIF-8@MXene crystals exhibited a regular shape with a rough and loose surface. The arrangement of tiny Fe-ZIF-8 wascompact packed on the inner and outer surface of the MXen. Figure 1c depicts the surface morphology of the Fe-ZIF-8@MXene, which was a layered structure, indicating successful preparation. After the adsorption of As(III) and As(V), the appearance of Fe-ZIF-8@MXene had obviously altered to become an extra dense and relatively smoother surface. Its original angularity and shape disappeared, which indicated that the outside and inside surfaces of the adsorbent had been covered with other particles, such as FeAsO_4_, hydroxides and As(III/V). Based of the scale of SEM, the particle size of Fe-ZIF-8@MXene is about 0.5–1.5 μm.

Figure 1c shows the XRD patterns of Fe-ZIF-8@MXene before and after adsorption. The main difference in XRD before and after adsorption was at 2θ of 11° and 38.9°. There were some new complexes from As(III) and As(V) interacting with a functional group of the adsorbent [36]. In addition, crystal phases of iron(III)-oxy/hydroxides were not found, indicating that iron likely existed in amorphous forms on this adsorbent [37]. It was reported that 2θ = 38.9° was the characteristic diffraction peak of FeAsO_4_, which similar phenomenon has been reported by Zang et al. [38]. The existence of the peak proved that Fe-ZIF-8@MXene had the potential ability to efficiently remove As(III) and As(V) combined pollution in wastewater. In addition, since the main peak locations hardly change and some peak intensities were only slightly increased or decreased after the adsorption, the crystal form and structure of Fe-ZIF-8@MXen had not significantly altered.

The As(III) and As(V) characteristics before and after adsorption were in the range of 4000–500 cm^−1^. There were two peaks at 2918 and 3127cm^−1^, which showed the C-H stretching vibration region on the saturated and unsaturated carbon, respectively. Peaks at 1658 and 1579 cm^−1^ are attributed to bending and stretching vibrations of N-H and imidazole, respectively [39,40]. After adsorption, there were two strong peaks at 821 and 816 cm^−1^, which resulted from O-As-Fe/Zn or O-As-N vibrations, and similar occurrence has been suggested by He et al. [41]. Therefore, it can be deduced that functional groups full of metal sites, nitrogen and oxygen may contribute to the adsorption of As species. 

The surface element information of Fe-ZIF-8@Mxene was acquired using XPS analysis.

**Figure 2:**
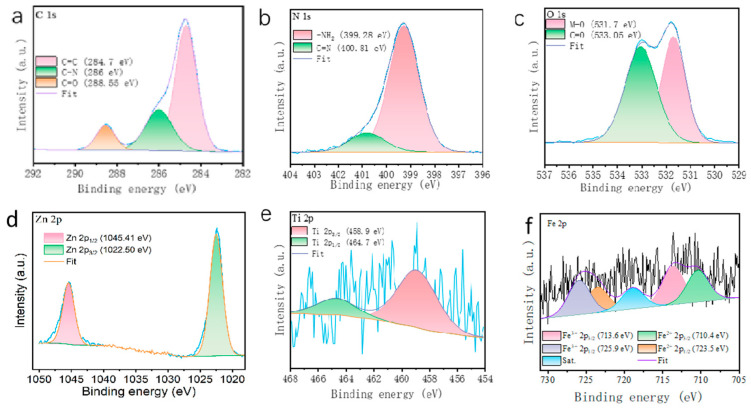
XPS images of C 1 s (**a**), N 1 s (**b**), O1s (**c**), Zn 2p (**d**), Ti 2p (**e**), and Fe 2p (**f**).

The spectrum shows a prominent peak at 284.7 eV (the pink line) (Figure 2a), attributable to C-C/C-H bonds. The peak at 286 eV (the green line), indicative of C-N bonds, highlights the presence of nitrogen-doped structures or organic ligands bonded to metal centers within the framework. The C=O peak at 288.55 eV (the orange line) points towards the presence of carboxylic groups or other oxygenated functionalities. In the N 1s spectrum (Figure 2b), the peak at 399.28 eV (the pink line) represents amine (-NH2) groups in the material, and the peak at 400.81 eV (the green line) could indicate metal–nitrogen coordination (C=N), further confirming the chemical environment of nitrogen in the material and potentially forming coordination bonds with metal ions.

The O 1s spectrum (Figure 2c) with peaks at 531.7 eV and 533.05 eV corresponds to metal-oxide (M-O) bonds and carbonyl C=O, respectively, indicating the presence of oxidized metal states and carboxyl groups on the material surface involved in interactions or adsorption processes with heavy metals. The Zn 2p spectrum (Figure 2d) shows two peaks at 1022.50 eV (the green line) and 1045.41 eV (the pink line), corresponding to Zn 2p3/2 and Zn 2p1/2, confirming the presence of Zn elements, consistent with the characteristics of ZIF-8. The peaks at 458.9 eV (the pink line) and 464.7 eV (the green line) correspond to metal Ti 2p3/2 and Ti 2p1/2, (Figure 2e), respectively. These groups are critical for the adsorption of pollutants through both physisorption and chemisorption mechanisms. The peaks at 710.4 eV (the green line) and 723.5 eV(the orange line) (Figure 2f) are attributed to Fe^2+^ in the 2p3/2 and 2p1/2 states, respectively. Similarly, the peaks at 713.6 eV (the pink line) and 725.9 eV (the light blue line) represent the Fe^3+^ in the 2p3/2 and 2p1/2 states. The black line represent general view of Fe. The presence of trivalent iron is indicative of the material’s role in adsorbing and stabilizing arsenic ions through complexation and electrostatic interactions, as Fe^3+^ typically forms more stable complexes with arsenate and arsenite, assisting in the immobilization of these contaminants. Moreover, the satellite peaks observed provide additional evidence of the complex chemical environment of iron within this material, highlighting its potential interaction with other compounds within the matrix. These interactions are pivotal for the effective trapping and stabilization of arsenic.

**Figure 3:**
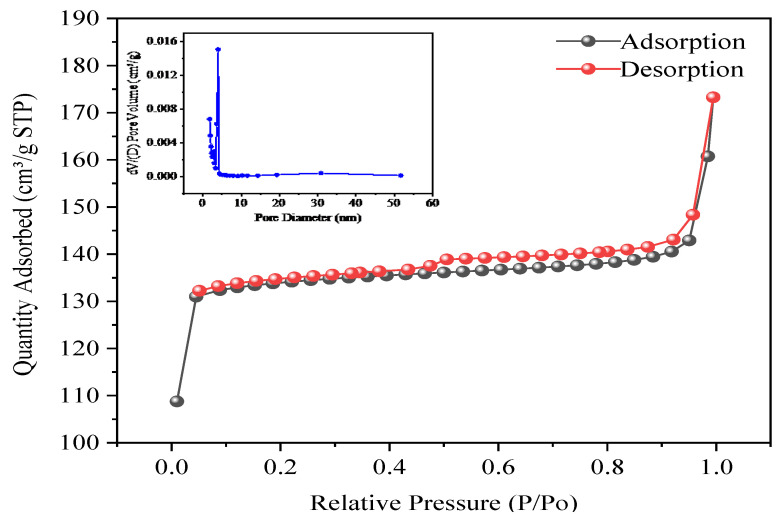
Nitrogen adsorption–desorption isotherm of Fe-ZIF-8-MXene and distribution of pore size.

Figure 3 shows the BET of Fe-ZIF-8@MXene based on the adsorption–desorption nitrogen isotherm at 77K. According to this study’s calculation, its BET was 531.7045 m^2^/g, the total pore volume was 0.223897 cm^3^/g and the average pore size was 1.6844 nm. This means that the adsorbent had a significantly porous structure. It was found to be a single layer adsorption at low pressure, which indicated the adsorption isotherms followed type I, according to the BDDT classification. The isotherm model illustrated that Fe-ZIF-8@MXene was mainly the gradient nanopore structure composed of the micropores and mesoporous pores. This finding further agreed with the results reported by Vithanage et al. [42]. The Langmuir equation could better explain the adsorption behavior, which was consistent with the following isotherm analysis.

The above results indicate the Fe-ZIF-8@MXene composites are synthesized, and the MXene keeps layered structure well during the process of hybridization.

### 3.2. Effect of Temperature and Concentration

**Figure 4:**
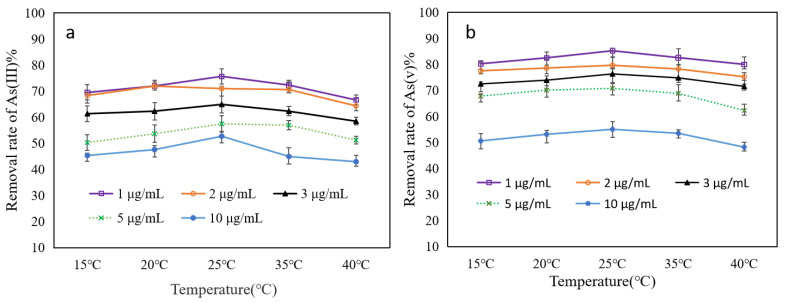
Effect of temperature and concentration on adsorption of As(III) (**a**) and As(V) (**b**).

With the temperature improved, the removal rate of both As(III) and As(V) also increased coherently. Higher temperatures could not only provide extra kinetic energy, but also diffuse As(V)/H_2_AsO_4_^−^ and As(III)/H_3_AsO_3_ in the solution. The increased temperature could also overcome the activation energy of the adsorbent. However, the removal rate only increased marginally with the increment in temperature from 15 to 25 °C (in Figure 4). After 25 °C, the removal rate of As(III) and As(V) started to slowed down. Generally, adsorption is an exothermic reaction, so a higher temperature does not contribute to the adsorption process by affecting the binding stability of As(III/V) with Fe-ZIF-8@MXene. Our preliminary findings also evidenced that a higher temperature could result in desorption by accelerating the movement of As(III) and As(V) off Fe-ZIF-8@MXene. Therefore, 25 °C was the turning point for the adsorption to be effective, which mainly accredited to partial desorption of As(III) and As(V) [43]. Notably, the removal rates of As(V) were always higher than those of As(III) at all temperature ranges for investigated experiments. The same rule of removal rate was also observed for the different concentrations of As(III) and As(V). Thus, the optimal treatment temperature was found to be 25 °C, and it was used for the subsequent adsorption tests.

The initial concentration of As(III/V) can significantly affect adsorption performance. The effect of five different initial mixture’s concentrations (1, 2, 3, 5 and 10 μg/mL) was examined with Fe-ZIF-8@MXene of 0.1 mg/L, at pH 5.8 at different temperatures. Overall, the removal rate of As(III) and As(V) swiftly decreased with the rise of the initial concentration of As(III/V) (Figure 4). For As(III)(Figure 4a), the removal rate remains slight decrease within the initial concentration of 1–3 μg/mL. However, the removal rate of As(III) significantly decreases when the initial concentration was 5–10 μg/mL. For As(V) (Figure 4b), the rule is similar to that of As(III). This was associated with the saturation of available binding sites. For a fixed concentration of adsorbent, its active sites and other functional groups were constant so that it could not adsorb the ever-increasing amount of As(III/V) presented in the solution. Therefore, with increased contaminant concentration for a constant Fe-ZIF-8@MXene dose, the removal efficiency gradually decreased.

### 3.3. Effect of pH

**Figure 5:**
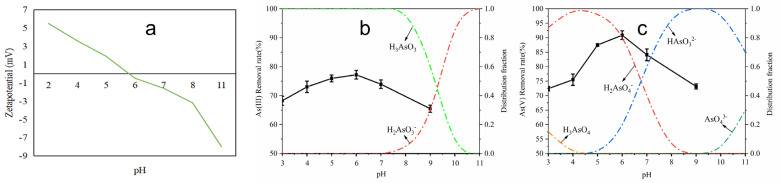
Effect of pH on adsorption of As(III) and As(V), zeta potential of Fe-ZIF-8@MXene ((**a**), the green line represent zeta potential), chemical species of As(III) ((**b**), the black line represent the removal rates of As(III) ) and As(V) ((**c**), the black line represent the removal rates of As(V)).

In principle, the solution’s pH plays an important role on the adsorbent and target pollutants, including ionization, solubility, hydrophilicity, surface charge and chemical speciation [44]. The change in pH drastically affects the removal rate of contaminants. Overall, considering the above factors, the impact of pH on As(III) and As(V) removal by Fe-ZIF-8@MXene was investigated between 3.0 and 9.0, as shown in Figure 5. Compared with As(V), the removal rate of As(III) was not visibly affected by pH change within the experimental range. In contrast, the adsorption of As(V) on Fe-ZIF-8@MXene apparently varied with the solution’s pH. With pH increased, the removal rate of As(V) rapidly raised, as was consistent with the electrostatic interactions. When pH was less than 5.8, As(III) and As(V) existed mainly as H_3_AsO_3_ and H_2_AsO_4_^-^/H_3_AsO_4_ in chemical species, respectively. Additionally, the zeta potential of Fe-ZIF-8@MXene was positively charged at pH < 5.8 (Figure 5a), which is beneficial to the adsorption of As(V) in the anionic state. While pH > 5.8, the zeta potential of Fe-ZIF-8@MXene was negatively charged, which disfavored the adsorption of the negative As anion species. From Figure 5b and c, when pH was equal to 5.8, the removal rates of As(III) and As(V) were at the maximum of 77.50% and 91.8%, respectively. The removal of As(V) was significant higher than that of As(III) since it formed stable complexes and/or co-precipitate iron arsenate [35]. As observed, metal Fe from Fe-ZIF-8@MXene reacted with As(V) to produce FeAsO_4_. Since over or under acidity could do harm to the environment and laboratory equipment, pH 5.8 has been used in all of the following adsorption tests.

### 3.4. Effect of Time and Adsorbent Dose on Adsorption Process

**Figure 6:**
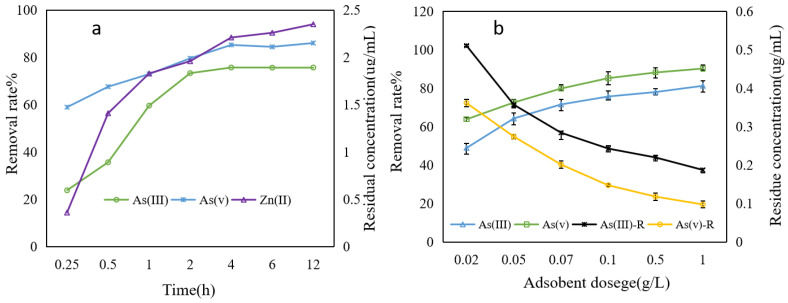
Effect of the adsorbent dosage on As(III) and As(V) removal with 1.0 μg/mL As(III/V) at pH 5.8 and room temperature. (**a**) and (**b**) are the effects of time and adsorbent on the removal rate of As(III/V), respectively.

The adsorption of As(III) and As(V) by Fe-ZIF-8@MXene can be roughly divided into three stages, and they are rapid adsorption (0–2 h), slow adsorption (2–4 h) and equilibrium stage (4–12 h) (Figure 6a). During the first adsorption stage, the slopes of the curve for both As(III) and As(V) were very high due to adequate adsorption sites and functional groups. During the second stage, most adsorption sites and available functional groups were occupied, resulting in the adsorption speed slowing down. In the last equilibrium stage, the removal rate was nearly as stagnant as that at 4 h. In addition, the concentration of Zn^2+^ in the solution gradually increased (even though it is relatively small) with the raising of the removal rate of As(III) and As(V) (Figure 6a). This phenomenon could be understood by some ion exchange reaction on the surface of the solid–liquid, with similar results also having been reported by Zhou et al. [45].

For the adsorbent dose, as logically expected, the removal rate increased with the higher dose of Fe-ZIF-8@MXene. Figure 6b shows that the removal rate of both As(III) and As(V) increased from approximately 48.92% and 63.84% to 81.26% and 90.25%, respectively, with the adsorbent dosage of Fe-ZIF-8@MXene increasing from 0.02 to 1.0 g/L, respectively. The removal rate of As(III) and As(V) increased quickly when the adsorbent dosage of Fe-ZIF-8@MXene was 0.02–0.1 g/L. The removal rate of As(III) and As(V) was slowly improved when the adsorbent dosage of Fe-ZIF-8@MXene was more than 0.1 g/L. With more adsorbent available, there are more adsorption sites to capture additional pollutant molecules. This outcome could be mainly linked to the increase in the number of exchangeable sites, BET and the possibility of interactions between Fe-ZIF-8@MXene particles and contaminants [46,47]. Nonetheless, excess adsorbent (more than 1g/L) will be treated as a kind of new waste.

### 3.5. Adsorption Kinetics and Isotherms

In order to further study the adsorption mechanism of Fe-ZIF-8@MXene on an As(III) and As(V) mixture solution, both pseudo-first-order and pseudo-second-order kinetics were explored to fit the kinetic data of the As(III) and As(V) adsorption processes, respectively.

The pseudo-first-order equation can be expressed as:

(3) $$\ln(q_{e}-q_{t})=\ln q_{e}-tk_{1}$$

(4) $$\frac{t}{q_{t}}=\frac{1}{q_{e}^{2}k_{2}}+\frac{t}{q_{e}}$$

In which *q_t_* is the adsorption capacity at time *t*, mg/g; *t* is the reaction time, min^−1^; *q_e_* is the equilibrium adsorption amount, mg/g; and *K*_1_ and *K*_2_ are the reaction rate constants of pseudo-first-order and pseudo-second-order kinetic equations, min^−1^ and g/(mg·min), respectively.

**Figure 7:**
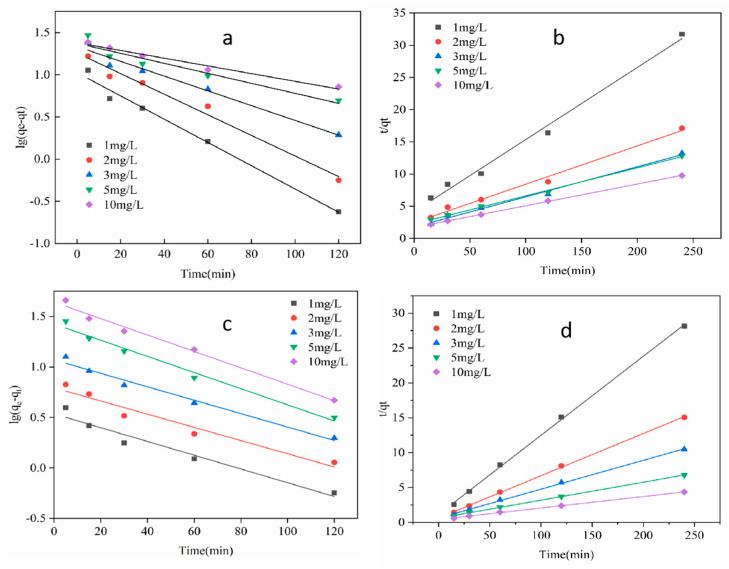
The pseudo-first/second-order rate model of As(III) and As(V) at pH 5.8 with 0.1 g/L Fe-ZIF-8@MXene and at room temperature. (**a**) and (**b**), the pseudo-first/second-order rate models of As(III), respectively, and (**c**) and (**d**), the pseudo-first/second-order rate models of As(V), respectively.

Kinetics is an important criterion because it not only determines the equilibrium time but also further dictates the adsorption mechanism. The kinetic experimental results are shown in Figure 7, and the parameters obtained from the kinetic model are listed in Table 1. For five samples with different initial concentrations of As(III) and As(V), R^2^ from the pseudo-second-order kinetic equation was all greater than 0.99, and which were also all greater than that from the pseudo-first-order equation. These demonstrated that the pseudo-second-order kinetic equation could better describe the adsorption kinetic processes. It further indicated that the chemisorption was the main step in the adsorption process. These finding confirmed that there were some chemical interactions on the surface of the solid–liquid between As ions and Fe-ZIF-8@MXene. The *q_e_* values of As(III) obtained from the pseudo-second-order equation model were 8.928–29.673 mg/g when the initial concentration of As(III) was 1–10 μg/mL. For As(V), *q_e_* was 8.818–60.606 mg/g for the initial concentration of As(V) between 1 and 10 μg/m, based on the pseudo second-order equation model. In addition, the equilibrium adsorption quantity *q_e_* of As(V) and As(III) is almost equal to their experimental data. These results indicated that the adsorption process was mainly chemical adsorption.

To further study the interaction mechanism of As(III) and As(V) with Fe-ZIF-8@MXene, adsorption isotherms were studied. The linear form of the Langmuir model considers monolayer adsorption on the surface of the adsorbent, where the equation can be written as

(5) $$\;\frac{c_{e}}{q_{e}}=\frac{1}{K_{L}q_{\max}}+\frac{c_{e}}{q_{\max}}$$

Furthermore, the Freundlich equation has been applied to describe an adsorption process on a reversible heterogeneous surface, where it can be shown as

(6) $$\;\ln q_{e}=\ln k_{F}+\frac{1}{n}\ln C_{e}\;$$

in which *Ce* is the equilibrium concentration of As(III) and As(V), mg/L; *q_e_* represents the equilibrium adsorption capacity of Fe-ZIF-8@MXene, mg/g; *q*_max_ is the maximum adsorption capacity of the Langmuir equation, mg/g; and *K_L_*, 1/n and *K_F_* are Langmuir and Freundlich constants, respectively.

**Figure 8:**
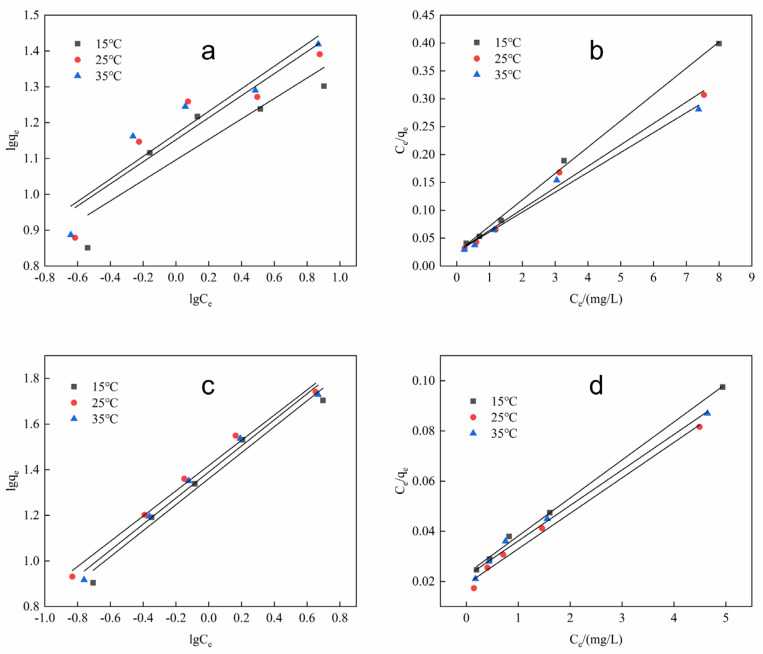
Langmuir and Freundlich adsorption isotherms for As(III) and As(V) with 1.0 μg/mL at pH 5.8 with 0.1 g/L Fe-ZIF-8@MXene and at room temperature. (**a**) and (**b**), the Langmuir and Freundlich adsorption isotherm of As(III), respectively, and (**c**) and (**d**), Langmuir and Freundlich adsorption isotherm of As(V), respectively.

The experimental results are shown in Figure 8, and the adsorption constants obtained from the Langmuir and Freundlich isotherm models at optimal conditions are listed in Table 2. The models of Langmuir (i.e., with the higher correlation coefficient) fitted well to represent experimental data. The adsorption capacities of As(III) and As(V) were 26.0417 and 70.9220 mg/g at 25 °C, respectively. In comparison to previous works [11,12,35], the proposed experimental result indicated that the adsorption capacities of As(III) and As(V) by Fe-ZIF-8@MXene have been improved remarkably. Therefore, this adsorbent is feasible for simultaneously removing As(III) and As(V) in the same solution.

### 3.6. Practical Application of Fe-ZIF-8@MXene in Authentic Wastewater

**Figure 9:**
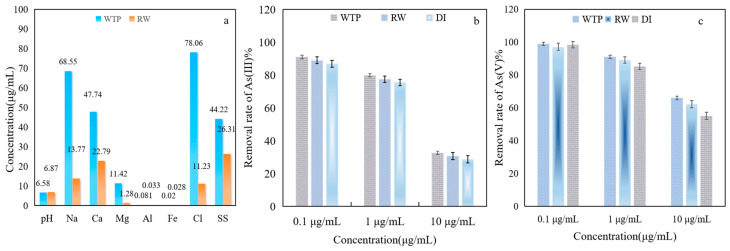
Effect of water samples from different sources on removal of As(III) and As(V) by Fe-ZIF-8@MXene ((**a**), the properties of WTP and RW and (**b**) and (**c**), removal of As(III) and As(V) in different water samples, respectively).

For practical purposes, it is imperative to perform the application not only on DI water but also on real water samples. To discuss the potential of Fe-ZIF-8@MXene to treat wastewater, a necessary experiment was carried out with real wastewater from different field sites. Three water samples were taken from RW, WTP and DI, respectively. In this test, As(III) and As(V) were added to wastewater (100 mL) with high, middle and low concentrations of As(III/V). They were used to treat As(III) and As(V) under the optimal condition, as per the results presented in Figure 9. 

The removal rate of As(III) was more than 80% when the concentration of As(III) was in the range of 0.1–1 μg/mL. For a high concentration of As(III) (≧10 μg/mL), its removal rate obviously decreased (<40%). For As(III) with 1 μg/mL, the removal rate increased from 75.7 % (in DI ) to 77.36 % (in RW) and to 79.94 % (in WTP) under the optimal condition. The order of removal rate in different solution samples is as follows: WTP ≥ RW ≥ DI no matter what the concentration of As(III/V). This is mainly due to the content of suspended solids (SS) in WTP being higher than that in RW (shown in Figure 9a). SS, which are one of the important standards by which to measure the quality of wastewater, have similar characteristics to tiny, suspended adsorbents. For the same source of sample, the higher concentration of As(III) and As(V) resulted in a lower removal rate under the same experimental conditions. The adsorption rule of As(V) is the same as that of As(III) except that the removal rate of As(V) was always higher than that of As(III). For As(V) with 1 μg/mL, the removal rate increased from 85.26% (in DI) to 88.97% (in RW) and to 91.02% (in WTP) under the optimal condition. The removal rate of As(V) for the three above-mentioned water samples was more than 96% (Figure 9c) when the concentration of As(V) was 0.1 μg/mL under the optimal parameters, which demonstrated that the residual As concentration As(V) could meet the discharge standards outlined in WHO (2001) [5].

### 3.7. Possible Mechanism for Adsorption

**Figure 10:**
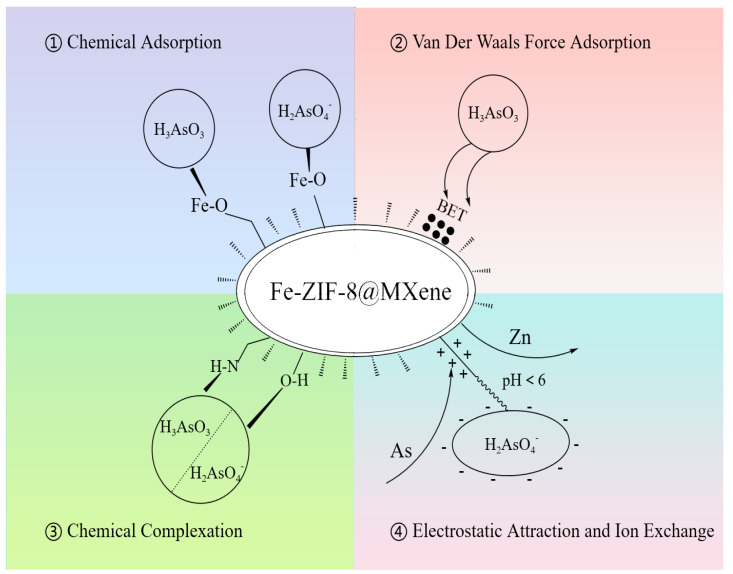
Possible mechanism for As(III) and As(V) adsorption.

A possible removal mechanism of As(III) and As(V) by Fe-ZIF-8@MXene was proposed after analysis of the presented experimental data (illustrated in Figure 10). Adsorption followed pseudo-second-order kinetics and was best-fitted by the Langmuir adsorption model. Based on pH and As species for As(III), the adsorption process on Fe-ZIF-8@MXene was mainly due to chemical complexation and Van der Waals force; however, for As(V), the adsorption process was mainly caused by ion exchange (As-OH^−^), chemical complexation (Fe-As) and electrostatic interactions (based on zeta potential-pH). The concentration of Zn^2+^ in the solution slowly rose with the increase in the removal rate of As(III) and As(V), which indicated that there was ion exchange during the adsorption process (Figure 6a). In addition, the surface morphology of Fe-ZIF-8@MXene changed greatly after adsorption, partly due to Zn^2+^ exchange from the adsorbent. Similar results could be seen in other studies [36,45].

## 4. Conclusions

Fe-ZIF-8@MXene was successfully prepared and applied as a perfect material to simultaneously remove both As(III) and As(V) from different real water sample sources. It was characterized by FTIR, XPS, XRD, TEM, SEM, BET surface area and Zeta potential. The results showed that Fe-ZIF-8@MXene maintained a stable structure after adsorption of As(III) and As(V). For both contaminants, the adsorption process followed pseudo-second-order kinetics which were best-fitted by the Langmuir adsorption model with correlation coefficients (R^2^) of 0.998 and 0.997 for As(III) and As(V), respectively. The maximum removal rate of As(III) and As(V) from real water RW and WTP (without any pretreatment) was 91.07% and 98.96%, respectively, under the optimal condition. Moreover, removal mechanisms for As(III/V) by Fe-ZIF-8@MXene were proposed. For As(III), the adsorption process on Fe-ZIF-8@MXene was primarily caused by chemical complexation and Van der Waals force. In comparison, the adsorption process for As(V) mainly depended on chemical complexation, electrostatic interactions and ion exchange. In summary, the residual concentration of As(V) could meet discharge standards for treatment of actual treated-water samples, and Fe-ZIF-8@MXene had a considerable role in the real application of the simultaneous removal of both As(III) and As(V) from aqueous solutions.

## Figures and Tables

**Table 1:** Kinetic parameters of As(III/V) adsorption by Fe-ZIF-8@Mxene.

Parameters	Pseudo-First-Order Model			Pseudo-Second-Order Model		
	*q_e_* (mg·g^−1^)	*K*_1_ (h^−1^)	R^2^	*q_e_* (mg·g^−1^)	*K*_2_ (g·mg^−1^·h^−1^)	R^2^
1 mg/LAs(III)	10.6047	0.0138	0.9876	8.9286	0.0030	0.9914
2 mg/L/As(III)	18.1301	0.0122	0.9833	16.8067	0.0013	0.9904
3 mg/L/As(III)	21.5874	0.0087	0.9722	21.3675	0.0012	0.9924
5 mg/L/As(III)	23.5993	0.0059	0.9207	22.9358	0.0008	0.9976
10 mg/L/As(III)	23.9552	0.0046	0.9781	29.6736	0.0007	0.9997
1 mg/L/As(V)	3.4261	0.0068	0.9550	8.8183	0.0112	0.9992
2 mg/L/As(V)	6.2158	0.0065	0.9523	16.5017	0.0058	0.9993
3 mg/L/As(V)	11.7382	0.0066	0.9787	24.1546	0.0027	0.9991
5 mg/L/As(V)	26.5705	0.0080	0.9824	38.7597	0.0011	0.9997
10 mg/L/As(V)	43.7925	0.0081	0.9870	60.6061	0.0007	0.9983

**Table 2:** Isotherms parameters of As(III) and As(V) adsorption by Fe-ZIF-8@MXene.

T (°C)/Adsorbates	Langmuir			Freundlich		
	*q*_max_ (mg/g)	*K_L_* (L/mg)	R^2^	*K_F_* (mg/g)	n	R^2^
15/As(III)	21.1864	1.9424	0.9980	12.4767	3.5002	0.8224
25/As(III)	26.0417	1.5000	0.9882	14.2004	3.2510	0.8652
35/As(III)	28.0899	1.4016	0.9884	14.7503	3.1786	0.8920
15/As(V)	65.7895	0.6609	0.9975	22.9245	1.7550	0.9733
25/As(V)	70.9220	0.7500	0.9918	26.1879	1.8067	0.9888
35/As(V)	79.4225	0.6455	0.9909	24.5358	1.7492	0.9853

## Data Availability

The data presented in this study are available from the corresponding author upon reasonable request.
